# Impact of Melatonin on RAW264.7 Macrophages during Mechanical Strain

**DOI:** 10.3390/ijms232113397

**Published:** 2022-11-02

**Authors:** Eva Paddenberg, Anne Forneck, Matthias Widbiller, Martyna Smeda, Jonathan Jantsch, Peter Proff, Christian Kirschneck, Agnes Schröder

**Affiliations:** 1 Department of Orthodontics, University Hospital Regensburg, 93053 Regensburg, Germany; 2 Department of Conservative Dentistry and Periodontology, University Hospital Regensburg, 93053 Regensburg, Germany; 3 Department of Microbiology and Hygiene, University Hospital Regensburg, 93053 Regensburg, Germany; 4 Institute for Medical Microbiology, Immunology and Hygiene, University Hospital Cologne and Faculty of Medicine, University of Cologne, 50935 Cologne, Germany

**Keywords:** macrophages, inflammation, melatonin, mechanical strain

## Abstract

The concentration of melatonin is elevated during the night when patients mainly wear removable orthodontic appliances. Next to periodontal ligament fibroblasts and osteoblasts, macrophages react to mechanical strain with an increased expression of inflammatory mediators. Here, we investigated the impact of melatonin on RAW264.7 macrophages exposed to tensile or compressive strain occurring during orthodontic tooth movement in the periodontal ligament. Before exposure to mechanical strain for 4 h, macrophages were pre-incubated with different melatonin concentrations for 24 h, to determine the dependence of melatonin concentration. Afterwards, we performed experiments with and without mechanical strain, the most effective melatonin concentration (25 µM), and the melatonin receptor 2 (MT2) specific antagonist 4P-PDOT. The expression of inflammatory genes and proteins was investigated by RT-qPCR, ELISAs, and immunoblot. Both tensile and compressive strain increased the expression of the investigated inflammatory factors interleukin-1-beta, interleukin-6, tumor necrosis factor alpha, and prostaglandin endoperoxide synthase-2. This effect was inhibited by the addition of melatonin. Incubation with 4P-PDOT blocked this anti-inflammatory effect of melatonin. Melatonin had an anti-inflammatory effect on macrophages exposed to mechanical strain, independent of the type of mechanical strain. As inhibition was possible with 4P-PDOT, the MT2 receptor might be involved in the regulation of the observed effects.

## 1. Introduction

The prevalence of malocclusions is 56% worldwide and 72% in Europe [1]. In addition to an enhanced prevalence of caries [2,3], tooth misalignments increase the prevalence of gingivitis, periodontitis, and recessions [4]. Orthodontic therapy can contribute to the prevention of these pathologies. Orthodontic tooth movement is induced by mechanical forces and leads to the formation of tension and compression zones in the periodontal ligament [5]. These mechanical strains provoke a sterile inflammatory reaction in the periodontal ligament and result in an increased release of proinflammatory enzymes, cytokines, and chemokines by periodontal ligament fibroblasts [6,7] and macrophages [8], affecting the extent of orthodontic tooth movement. The orthodontic force is transmitted to the tooth either by fixed or removable appliances. The compliance of patients to wear removable appliances is greater at night when the release of the endogenous hormone melatonin is increased [9,10].

In mammals melatonin can act by different mechanisms like antioxidant effects, binding to intracellular proteins like calmoduline or via melatonin receptors in the plasma membrane [11,12,13,14,15]. Three types of melatonin receptor have been recognized and classified as MT1, MT2 and MT3 [16,17]. Next to its anti-inflammatory properties [12,13,14], melatonin is necessary for circadian rhythm. Due to rapid breakdown and decreased production, melatonin levels are lower during the day than at night [18,19]. Melatonin synthesis oscillates due to signals from the suprachiasmatic nucleus. The melatonin that is measurable in the saliva [20,21] originates from the pineal gland. In addition to the pineal gland, extrapineal production sites such as the retina, intestinal mucosa, liver, kidney, thymus, thyroid, mast cells, leukocytes, endothelial cells, skin and other tissues have been identified [22,23,24,25]. Melatonin can impact on bone remodeling processes by upregulation of the expression of bone-forming proteins [26,27,28,29,30,31,32] and downregulation of osteoclastogenesis [33]. As bone remodeling plays a crucial role in orthodontic movement, melatonin could have a mediating role in the orthodontic movement of teeth.

Mechanical stress increases the expression of prostaglandin endoperoxide synthase-2 (PTGS2) in periodontal ligament fibroblasts [6,7] and macrophages [8], which promotes the synthesis of prostaglandin E2 (PGE2). PGE2 induces receptor activator of NF-kB ligand (RANKL) expression in periodontal ligament fibroblasts [7], which is crucial for osteoclastogenesis. Osteoclastogenesis, however, is also mediated by tumor necrosis factor alpha (TNFα) [34,35]. Therefore, inflammatory cytokines are also factors that contribute to the regulation of osteoclastogenesis. These could be affected by the anti-inflammatory properties of melatonin.

Since melatonin is detectable in saliva and removable orthodontic appliances are mainly worn at night, it could impact on macrophages exposed to mechanical strain and impair their proinflammatory response. In this basic-research-based study, therefore, we aimed to investigate the possible influence of melatonin on RAW264.7 macrophages during mechanical strain. Therefore, we hypothesize that melatonin affects the reaction of macrophages to mechanical strain.

## 2. Results

### 2.1. Determination of the Concentration Dependence of Melatonin on Macrophages during Mechanical Strain

**Figure 1:**
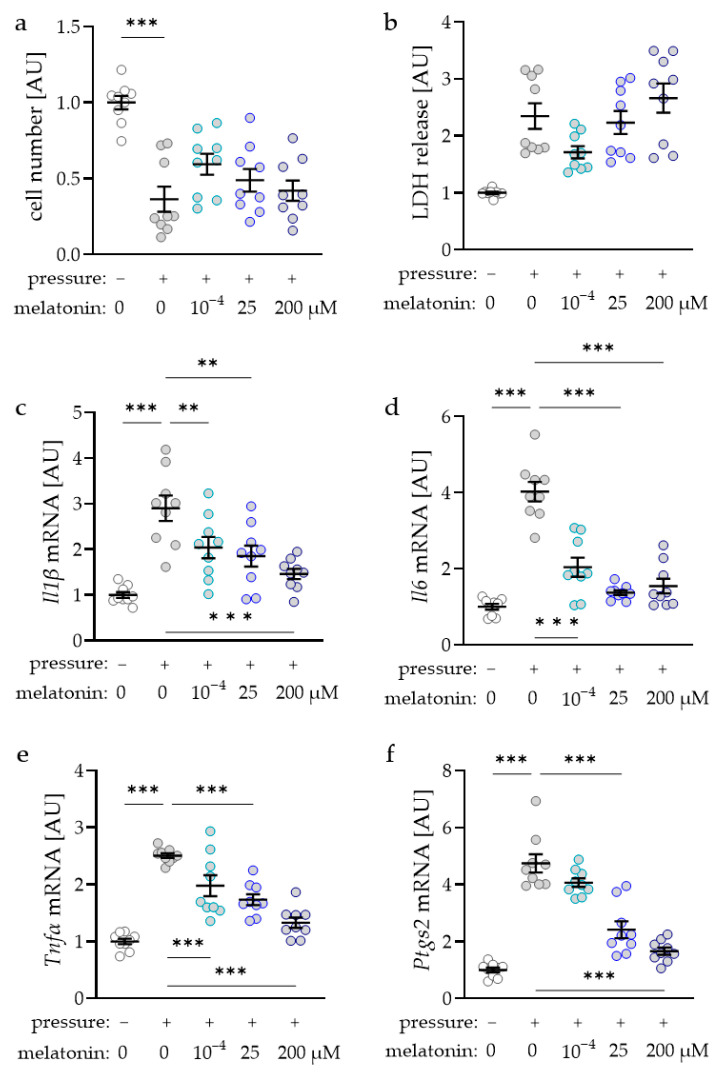
Impact of different melatonin concentrations in combination with compressive strain on cell number (**a**), lactate dehydrogenase (LDH) release (**b**) and gene expression of interleukin-1-beta (IL1β, (**c**)), IL6 (**d**), tumor necrosis factor alpha (TNFα, (**e**)), or prostaglandin endoperoxide synthase-2 (PTGS2, (**f**)); *n* = 9; Statistics: ordinary ANOVA with Holm–Šídák’s multiple comparison tests (*Il1β*, *Il6*, and *Tnfα*) or Welch-corrected ANOVA with Games–Howell multiple comparison tests (cell number, LDH release, *Ptgs2*); ** *p* < 0.01, *** *p* < 0.001.

First, we determined the concentration dependence of melatonin for the subsequent experiments. Cell number decreased in reaction to compressive strain under control conditions (*p* < 0.001; Figure 1a). No effects of the tested melatonin concentrations were detected on the determined cell number (*p* > 0.250) compared to the compressed control without melatonin. Accordingly, lactate dehydrogenase (LDH) release was upregulated after compression (*p* = 0.002) with no additional melatonin effects (*p* > 0.147; Figure 1b). Compressive force increased interleukin-1-beta (*Il1ß*) gene expression under control conditions without additional melatonin (*p* < 0.001; Figure 1c). However, the pressure effect was mitigated in reaction to the addition of melatonin (*p* < 0.004; Figure 1c). Gene expression of *Il6* was also upregulated after compression (*p* < 0.001; Figure 1d). Again, all tested melatonin concentrations reduced this pressure-induced increase in *Il6* gene expression (*p* < 0.001). Gene expression of tumor necrosis factor alpha (*Tnfα*) was elevated with compression (*p* < 0.001; Figure 1e). Treatment with all tested melatonin concentrations reduced this effect significantly (*p* < 0.001). Analysis of prostaglandin endoperoxide synthase-2 (*Ptgs2*) gene expression revealed a significant induction after pressure application (*p* < 0.001; Figure 1f). Only treatment with 25 µM or 200 µM melatonin (*p* < 0.001) reduced this effect of compressive strain significantly, while this effect was not visible after addition of 10^−4^ µM melatonin (*p* = 0.367; Figure 1f).

### 2.2. Impact of Melatonin on Expression of Inflammatory Genes during Tensile Strain

**Figure 2:**
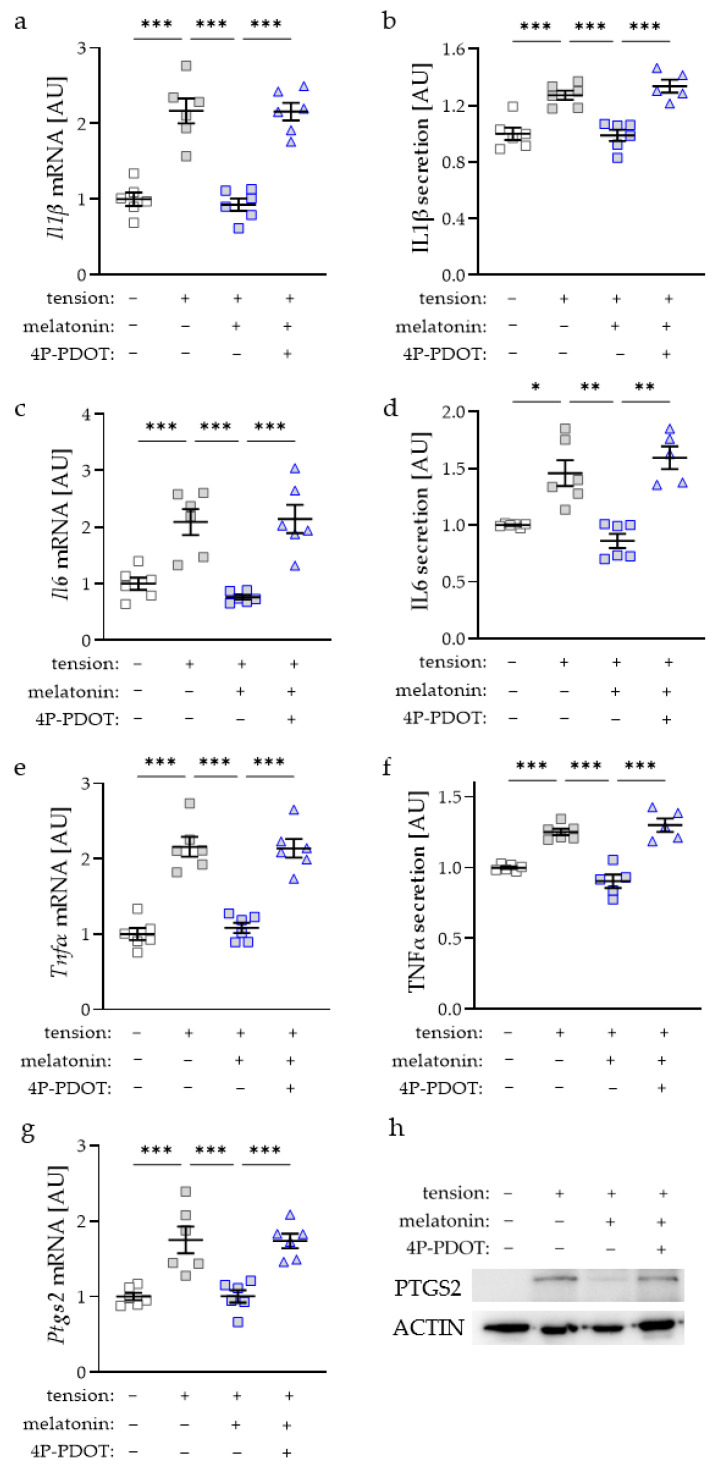
Impact of melatonin and tensile strain on gene and protein expression of interleukin-1-beta (IL1β; (**a**,**b**)), IL6 (**c**,**d**), tumor necrosis factor alpha (TNFα, (**e**,**f**)), or prostaglandin endoperoxide synthase-2 (PTGS2, (**g**,**h**)) in macrophages; uncropped blot is presented in Figure S1. *n* ≥ 5; Statistics: ordinary ANOVA with Holm–Šídák’s multiple comparison tests expect IL6 secretion (Welch-corrected ANOVA with Games–Howell multiple comparison tests); * *p* < 0.05, ** *p* < 0.01, *** *p* < 0.001.

Tensile strain increased *Il1β* gene expression (Figure 2a) and IL1β secretion (Figure 2b) under control conditions without additional melatonin (*p* < 0.001). Treatment with melatonin inhibited this tensile-strain-induced effect at the mRNA and protein levels (*p* < 0.001). The addition of melatonin receptor antagonist 4P-PDOT restored the strain-induced increase of the *Il1β* gene and protein expression (*p* < 0.001; Figure 2a,b). Analysis of *Il6* showed comparable results. Tensile-strain-induced upregulation of *Il6* gene expression (*p* < 0.001) was inhibited by the addition of melatonin (*p* < 0.001; Figure 2c). Again, a combination of melatonin with 4P-PDOT treatment led to an elevated *Il6* gene expression (*p* < 0.001). The same was detectable at the protein level (Figure 2d). Gene expression and secretion of TNFα were increased with tensile strain (*p* < 0.001; Figure 2e,f). This effect was inhibited by melatonin addition (*p* < 0.001). Inhibition of melatonin receptor by 4P-PDOT showed an inducible effect of tensile strain on TNFα (*p* < 0.001; Figure 2e,f). PTGS2 was upregulated with tensile strain at the mRNA (*p* < 0.001) and protein level (Figure 2g,h). This tension-induced effect was again inhibited by the addition of melatonin (*p* < 0.001; Figure 2g). 4P-PDOT restored the effect of tensile strain on *Ptgs2* gene expression (*p* < 0.001; Figure 2g).

### 2.3. Impact of Melatonin on Expression of Inflammatory Genes and Proteins during Compressive Strain

**Figure 3:**
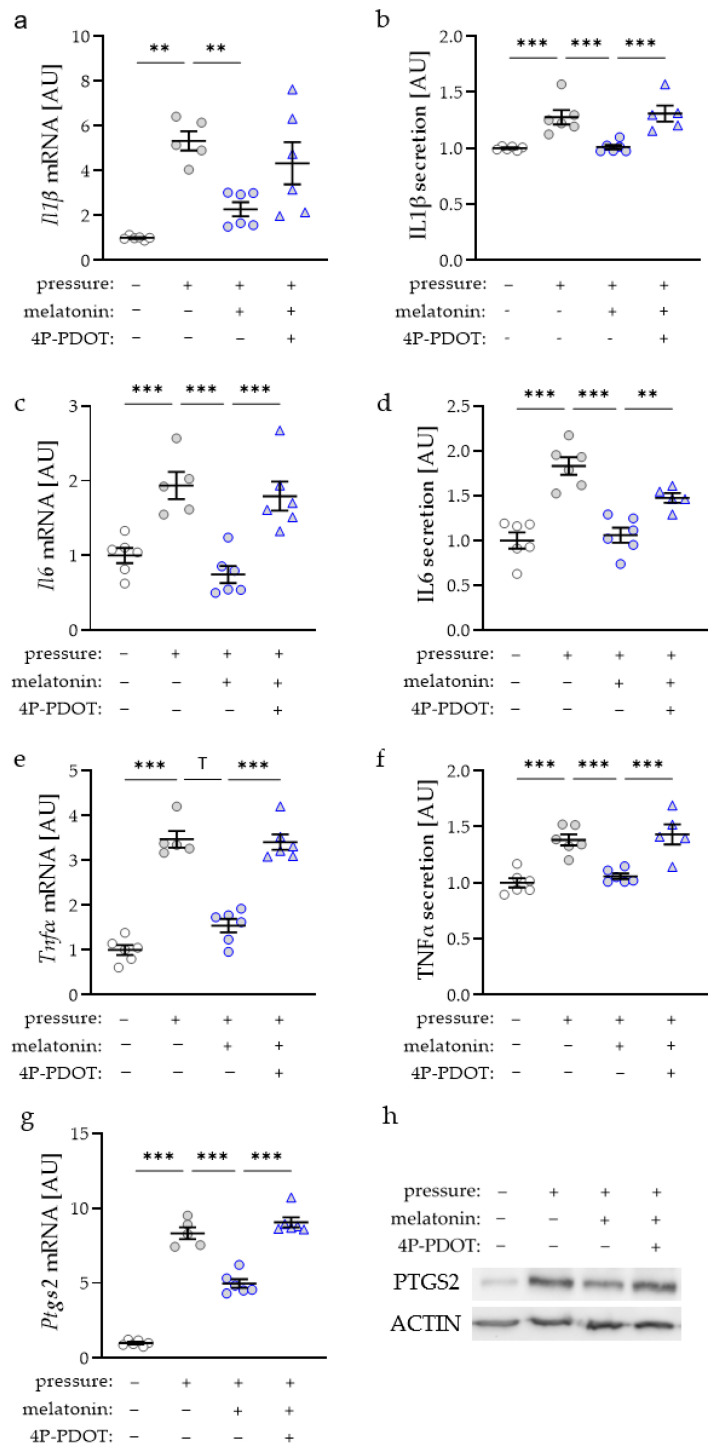
Impact of melatonin and compressive strain on gene and protein expression of interleukin-1-beta (IL1β; (**a**,**b**)), IL6 (**c**,**d**), tumor necrosis factor alpha (TNFα, (**e**,**f**)), or prostaglandin endoperoxide synthase-2 (PTGS2, (**g**,**h**)) in macrophages; uncropped blot is presented in Figure S2. *n* ≥ 5; Statistics: ordinary ANOVA with Holm–Sidak’s multiple comparison tests except for *Il1β*, *Tnf*α and *Ptgs2* mRNA (Welch-corrected ANOVA with Games–Howell multiple comparison tests); ^T^ *p* < 0.10; ** *p* < 0.01, *** *p* < 0.001.

Like tensile strain, pressure application increased *Il1β* gene expression (*p* = 0.004; Figure 3a). Addition of 25 µM melatonin inhibited this compressive-strain-induced upregulation of *Il1β* gene expression (*p* = 0.007). On the protein level, we observed an increased IL1β secretion after compression (*p* = 0.002; Figure 3b). According to the mRNA level, this was reduced after melatonin treatment (*p* = 0.003). When melatonin was antagonized with 4P-PDOT, the induction of IL1β secretion due to compressive strain was restored (*p* = 0.001; Figure 3b). IL6 gene expression (*p* = 0.038) and secretion (*p* < 0.001) were elevated with compressive strain (Figure 3c,d). Again, melatonin inhibited the pressure-induced induction of IL6 mRNA (*p* = 0.011) and protein secretion (*p* < 0.001). The addition of 4P-PDOT to melatonin-treated cells restored the effect of compressive force on the IL6 gene and protein secretion (Figure 3c,d). Similar effects of melatonin and the combination of melatonin with 4P-PDOT were observed for TNFα gene expression (*p* = 0.067, Figure 3e) and secretion (*p* < 0.001, Figure 3f). As expected, PTGS2 was upregulated with compressive strain at the mRNA (*p* < 0.001, Figure 3g) and protein level (Figure 3h). This effect on PTGS2 expression was inhibited by the addition of melatonin. When melatonin was combined with 4P-PDOT, the pressure-induced increase of PTGS2 expression was restored (Figure 3g,h).

## 3. Discussion

To evaluate the role of melatonin on macrophages during mechanical strain, we started this study with an evaluation of the melatonin concentration for the experiments with mechanical strain. Melatonin was determined in saliva samples in concentrations ranging from 10^−6^ during the day to 10^−4^ µM at night [19,20,21]. Other studies conducted with macrophages used higher melatonin concentrations ranging up to 1 mM [36,37,38,39]. With the physiological concentration of 10^−4^ µM melatonin, we already detected effects on proinflammatory gene expression in RAW264.7 macrophages. With a concentration of 25 µM melatonin, all tested proinflammatory genes were affected with no cytotoxic effects of melatonin. Therefore, we decided to use this concentration for the experiments with mechanical strain.

Orthodontic tooth movement can be attributed to the action of various chemokines and cytokines secreted by cells in the periodontal ligament exposed to mechanical strain. These cells are exposed to compressive and tensile forces when an orthodontic force is applied to the tooth to be moved in a specific direction. Next to periodontal ligament fibroblasts, which constitute the main cell population in the periodontal ligament, immune cells such as macrophages can modulate the local sterile immune response [35] during orthodontic tooth movement.

According to previous studies, we detected increased expression of proinflammatory cytokines like tumor necrosis factor (TNF), prostaglandin endoperoxide synthase-2 (PTGS2), and interleukins (IL1β, IL6) with mechanical strain [8], indicating a shift to M1-like polarization. This enhanced production of cytokines could impact the extent of orthodontic tooth movement. In combination with melatonin, we detected an anti-inflammatory effect on macrophages exposed to mechanical strain, independent of the type of mechanical strain.

Several studies on the effect of melatonin on macrophages have already been conducted, but none in combination with mechanical strain. Human and mouse macrophages treated with melatonin showed a reduction of the inducible nitric oxide synthase [38] and TNF [40]. RAW264.7 macrophages stimulated with lipopolysaccharides or fimbriae of bacteria were shown to decrease the expression of inflammatory cytokines (IL1β, IL6, TNFα, and PTGS2) with different melatonin treatments up to 1 mM [36,37,39,41], which is in line with our data. Most observed effects were blocked with the MT2-specific antagonist 4P-PDOT [42], indicating that effects on inflammatory factors are mediated by this receptor. To prove a mediation of these effects by MT2, however, further experiments would have to be conducted as melatonin can also exert receptor independent regulations [24].

Indeed, studies showed that high local concentrations of melatonin or its metabolites mediate their effects through mitochondria or activation of nuclear receptors [24,25]. Moreover, since melatonin is rapidly metabolized, some effects could also be mediated by the metabolites [24,43]. In mammals melatonin can act by four different mechanisms: antioxidant effects, binding to orphan nuclear receptors, to intracellular proteins like calmoduline or to melatonin receptors in the plasma membrane [11,12,13,14,15]. Macrophages were known to express melatonin receptor 1 and 2 (MT1, MT2) but also shown to produce melatonin by themselves [44,45,46]. The MT2 receptor can act through a number of signal transduction pathways. These include phosphoinositide production, inhibition of adenylyl cyclase and of soluble guanylyl cyclase pathway [13,47]. A possible mechanotransductive pathway and signaling could involve phosphorylation of kinases like ERK [48,49,50]. Melatonin can also affect the downstream effects of Ca^2+^ signaling as it directly antagonizes binding of Ca^2+^ to calmoduline [15,48,51] which might also play a role in mechanotransduction via mechanosensitive PIEZO channels [52].

Recently, periodontal ligament fibroblasts were shown to express MT1 and MT2 [53]. Contrary to macrophages, melatonin further elevated the effect of mechanical strain on the expression of inflammatory cytokines in periodontal ligament fibroblasts [53]. This could compensate for the reduction in cytokine production by macrophages in vivo.

This study has several limitations which should also be addressed. This in vitro study was performed using RAW264.7 macrophages to investigate the effect of melatonin and mechanical strain on macrophages. RAW264.7 macrophages were often used for in vitro experiments in basic research as model cell lines representing macrophages, but the reaction of tissue-resident macrophages might be different. In a previous study, the effects of mechanical stress on RAW264.7 macrophages could also be reproduced in bone marrow-derived macrophages [8], but no data are available from macrophages derived from periodontal ligament. In this basic research study, the RAW264.7 macrophages were subjected to compressive and tensile strain using well-established and accepted in vitro models in orthodontic research [7,54]. Both mechanical strains occur in the periodontal ligament after a force is applied to the tooth being moved. However, mechanical forces may not only occur during orthodontic movement but also act, e.g., in swollen lymph nodes or tumors due to hydrostatic pressure well. Furthermore, this basic study is limited to one cell type that occurs in the periodontal ligament. Next to macrophages, periodontal ligament fibroblasts, osteoblasts, and leukocytes could be affected by melatonin. As melatonin is a hormone that affects the whole body and orthodontic tooth movement is a multicellular process depending on the communication of several cell types, in vivo studies should be performed to further unravel the effect of melatonin on orthodontic tooth movement.

Our data demonstrated an anti-inflammatory effect of melatonin on macrophages exposed to compressive and tensile mechanical strain. As inhibition was possible with 4P-PDOT, the MT2 receptor might be involved in the regulation of the observed effects.

## 4. Materials and Methods

### 4.1. Cell Culture Experiments

RAW264.7 macrophages (Cell Lines Service, Eppelheim, Germany) were cultured in Dulbecco’s Modified Eagle Medium (41966029, Thermo Fisher Scientific, Waltham, MA, USA) supplemented with 10% FBS (P30-3302, PAN-Biotech, Aidenbach, Germany) and 1% antibiotic/antimycotic (A5955, Sigma Aldrich, St. Louis, MO, USA).

**Figure 4:**
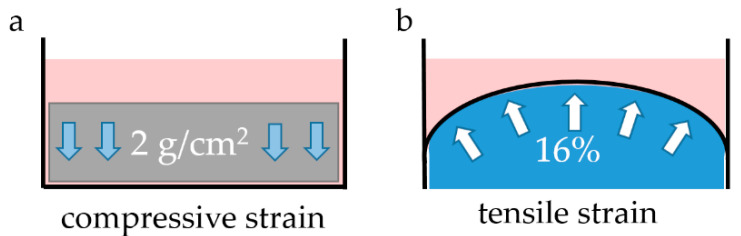
Schematic representation of the application of static pressure (2 g/cm^2^, (**a**)) and tensile strain (16%, (**b**)) for 4 h.

Experiment 1: To determine the most effective melatonin concentration, 10^6^/well RAW264.7 cells were seeded on 6-well cell culture plates (353046, Omnilab, Bremen, Germany) and pre-incubated with different melatonin concentrations (10^−4^ µM, 25 µM; 200 µM; M5250, Sigma Aldrich, St. Louis, MO, USA) for 24 h. Melatonin was dissolved in ethanol to a final concentration of 200 mM. Subsequent dilutions were performed in medium. Pressure was applied for 4 h to the cells using glass plates (2 g/cm^2^) as already described (Figure 4a) [8]. Afterwards, RNA was isolated and gene expression was analyzed with RT-qPCR. Based on the results, a melatonin concentration of 25 µM was used for the following experiments.

Experiment 2: To apply isotropic tensile strain, 10^6^ RAW264.7 macrophages per well were seeded on BioFlex culture plates (BF-3001U, Dunn Labortechnik, Asbach, Germany). The cells were either left untreated, or treated with 25 µM melatonin (M5250, Sigma Aldrich, St. Louis, MO, USA) in combination with 100 nM 4P-PDOT (1034, Tocris Bioscience, Bristol, UK) for 24 h. After the pre-incubation period, the cells were exposed to tensile strain using spherical silicone stamps (16%) as previously described (Figure 4b) [8,54] or left untreated for another 4 h. Cell number and cytotoxicity (Figure S3) as well as gene and protein expression were analyzed.

Experiment 3: For compressive strain, 10^6^ RAW264.7 macrophages per well on conventional 6-well cell culture plates (353046, Omnilab, Bremen, Germany) and either pre-incubated with 25 µM melatonin (M5250, Sigma Aldrich St. Louis, MO, USA) in combination with 100 nM 4P-PDOT (1034, Tocris Bioscience, Bristol, UK) for 24 h or left untreated. Pressure was applied for 4 h to the cells using glass plates (2 g/cm^2^) as already described (Figure 4a) [8]. Cell number and cytotoxicity (Figure S4) as well as gene and protein expression were analyzed.

### 4.2. Determination of Cell Number

Cells were removed in 1 mL of PBS using a cell scraper. 100 µL of the cell suspension was added to 10 mL of 0.8% sodium chloride solution and quantified using a Coulter Counter (Z2, Beckham Coulter, Brea, CA, USA). For quantification, a cell size of 8–15 µm was set.

### 4.3. Lactate Dehydrogenase (LDH) Assay

An LDH assay (04744926001, Roche, Penzberg, Germany) was performed according to the manufacturer’s instructions to assess cytotoxicity.

### 4.4. RNA Isolation

Cells were incubated according to the mentioned protocols. The cell culture supernatant was removed, and the cells were scraped from the bottom of the 6-well plates in 1 mL PBS using a cell scraper. The cell suspension was centrifuged at 2000 rpm for 5 min at 4 °C. The cell pellet was dissolved in 500 µL RNA Solv Reagent (R6830-01, WR international, Radnor, PA, USA). After the addition of 100 µL chloroform, the samples were vortexed for 30 s. Samples were incubated on ice for 10 min and were then centrifuged at 13,000 rpm for 15 min at 4 °C. The resulting aqueous supernatant was transferred to a fresh tube containing 500 µL isopropanol (20.842.330, WR international, Radnor, PA, USA). The samples were incubated overnight at −80 °C and centrifuged at 13,000 rpm for 30 min at 4 °C followed the next day. The supernatant was discarded and the pellet was washed twice with 500 µL 80% ethanol. After centrifugation at 13,000 rpm for 10 min at 4 °C, the supernatant was removed and the samples were dried for 30 min. The pellet was resuspended in 20 µL nuclease-free water (T143.5, Carl Roth, Karlsruhe, Germany). The purity and amount of total RNA were measured photometrically at 260 nm and 280 nm (N60, Implen, Munich, Germany).

### 4.5. cDNA Synthesis

For reverse transcription, equal concentrations of RNA were diluted with nuclease-free water (T143.5, Carl Roth, Karlsruhe, Germany) to a volume of 5.5 µL. To each sample, 2 µL of M-MLV reverse transcriptase buffer (M1705, Promega, Madison, WI, USA), 0.5 µL of OligodT (SO132, Thermo Fisher Scientific, Waltham, MA, USA), 0.5 µL of random hexamer primer (SO142, Thermo Fisher Scientific, Waltham, MA, USA), 0.5 µL of dNTPs (L785. 2, Carl Roth, Karlsruhe, Germany), 0.5 µL RNAse inhibitor (EO0382, Thermo Fisher Scientific, Waltham, MA, USA), and 0.5 µL reverse transcriptase (M1705, Promega, Madison, WI, USA) were added. To avoid errors due to pipetting, the mixture was prepared as a master mix for the entire experimental approach, so 4.5 µL of the master mix was added to 5.5 µL of the adjusted RNA sample. After mixing, samples were incubated at 37 °C for 1 h, followed by inactivation at 95 °C heat for 2 min and stored at 4 °C.

### 4.6. Quantitative Real-Time Polymerase Chain Reaction (RT-qPCR)

A reaction mix was prepared for each gene to be tested (Table 1). For 8.5 µL of mix per well, 0.25 µL each of the forward primer, 0.25 µL of the reverse primer, 5 µL of Luna Universal qPCR Master Mix (M3003E, New England BioLabs, Ipswich, MA, USA), and 3 µL of nuclease-free water (T143.5, Carl Roth, Karlsruhe, Germany) were mixed. 96-well plates (712282, Biozym, Hessisch Oldendorf, Germany) were used. Each sample and gene were analyzed in duplets. For this, 8.5 µL of the primer mix was mixed with 1.5 µL of the cDNA solution in each well using an electronic multipette (Multipette stream, Eppendorf) to minimize variations due to pipetting. The plates were sealed with a transparent film (712350, Biozym, Hessisch Oldendorf, Germany). A combination of the housekeeping genes *Eef1a1/Sdha* for pressure experiments and *Gapdh/Tbp* for the experiments with tensile strain was used as a reference (Table 1) [8].

The plate was placed in the Mastercycler ep Realplex-S Thermocycler (Eppendorf, Hamburg, Germany). After 2 min of heat activation at 95 °C, the program went through cycles in which the steps of 10 s denaturation at 95 °C, 20 s attachment at 60 °C, and 8 s elongation at 72 °C were repeated a total of 45 times. At the end of each cycle, fluorescence was quantified.

### 4.7. Western Blot Analysis

Protein was isolated with 100 µL of CelLytic (C2978, Sigma Aldrich, St. Louis, MO, USA) supplemented with proteinase inhibitor (87786, Thermo Fisher Scientific, Waltham, MA, USA) and concentration was determined with RotiQuant (K015.3, Carl Roth, Karlsruhe, Germany) according to manufacture’s instructions. Equal amounts of protein were separated on 10% polyacrylamid gels. The proteins were transferred to a PVDF membrane with a pore size of 0.45 µm (T830.1, Carl Roth, Karlsruhe, Germany) for 90 min at 90 V. To prevent subsequent nonspecific binding of antibodies to the membrane, the blotted membrane was incubated for 1 h at room temperature in 5% milk powder (T145.3, Carl Roth, Karlsruhe, Germany) dissolved in Tris-buffered saline with Tween20 (TBS-T). ACTIN (E1C602-2, EnoGene, New York, NY, USA) and PTGS2 (PA5-16817, Thermo Fisher Scientific, Waltham, MA, USA) were used as primary antibodies. These were each incubated overnight at 4 °C and after washing three times in TBS-T, the membrane was incubated in the secondary antibody (611-1302, Rockland Immunochemicals, Gilbertsville, PA, USA) for 1 h at room temperature. After washing in TBS-T, Luminata Crescendo Western HRP Substrate (WBLUR0100, Sigma Aldrich, St. Louis, MO, USA) was added and the signal was digitized using the VWR Genoplex documentation system (VWR international, Radnor, PA, USA).

### 4.8. Enzyme-Linked Immunosorbent Assay (ELISA)

Culture supernatants were stored at −80 °C and thawed for the ELISA assays. ELISAs were performed for murine interleukin-1-beta (IL1β, MBS412296, MyBiosource, San Diego, CA, USA), tumor necrosis factor alpha (TNFα, MBS335449, MyBiosource, San Diego, CA, USA), and interleukin-6 (IL6, MBS335514, MyBiosource, San Diego, CA, USA). The kits were used according to the manufacturer’s instructions.

### 4.9. Statistical Methods

Horizontal lines in figures show mean values, the vertical line represents the standard error of the mean, and symbols show individual data points. The Shapiro–Wilk test was used to check for the normal distribution of data. Depending on the normal distribution, either a Welch–corrected ANOVA with a Games–Howell’s multiple comparison or an ANOVA with Holm-Sidak’s multiple comparison was performed. We used GraphPad Prism Version 9.0 (GraphPad software, San Diego, CA, USA) for statistical analysis and rated data as statistically significant at *p* < 0.05. All data were presented as fold induction and named arbitrary units [AU]. For this purpose, all data were divided by the calculated mean value of the respective control group. For the controls, this resulted in a fold induction of 1.

## Figures and Tables

**Table 1:** Primer sequences for reference genes (pressure, *Eef1a1/Sdha*; tension, *Gapdh/Tbp*) and target genes.

Symbol	Gene Name	Forward Primer	Reverse Primer
*Eef1a1*	Eukaryotic translation elongation factor-1-alpha-1	AAAACATGATTACAGGCACATCCC	GCCCGTTCTTGGAGATACCAG
*Gapdh*	Glyceraldehyde-3-phosphate dehydrogenase	GTCATCCCAGAGCTGAACGG	ATGCCTGCTTCACCACCTTC
*Il1β*	Interleukin-1-beta	GTGTAATGAAAGACGGCACACC	ACCAGTTGGGGAACTCTGC
*Il6*	Interleukin 6	AAAGCCAGAGTCCTTCAGAGAG	CCTTAGCCACTCCTTCTGTGAC
*Ptgs2*	Prostaglandin-endoperoxide-Synthase 2	TCCCTGAAGCCGTACACATC	TCCCCAAAGATAGCATCTGGAC
*Sdha*	Succinate dehydrogenase complex, subunit A	AACACTGGAGGAAGCACACC	AGTAGGAGCGGATAGCAGGAG
*Tbp*	TATA box binding protein	CTATCACTCCTGCCACACCAG	CACGAAGTGCAATGGTCTTTAGG
*Tnfα*	Tumor necrosis factor alpha	ACAAGCCTGTAGCCCACGTC	TTGTTGTCTTTGAGATCCATGCC

## Data Availability

All datasets are publicly available either as supplementary information to this article or upon request from the corresponding author.

## Supplementary Materials

The following supporting information can be downloaded at: https://www.mdpi.com/article/10.3390/ijms232113397/s1.
