# Supplementary material for: Impact of Melatonin on RAW264.7 Macrophages during Mechanical Strain
Source: Int J Mol Sci. 2022 Nov 2;23(21):13397. doi: 10.3390/ijms232113397 (PMC9655379; doi:10.3390/ijms232113397)
Supplement: Supplementary file 1 [file ijms-23-13397-s001.zip › ijms-1942335-supplementary.pdf]

Article

Impact of Melatonin on RAW264.7 Macrophages during Mechanical Strain

Eva Paddenberg [1], Anne Forneck [1], Matthias Widbiller [2], Martyna Smeda [2], Jonathan Jantsch [3,4], Peter Proff [1], Christian Kirschneck [1,†] and Agnes Schröder [1,3,*,†]

1 Department of Orthodontics, University Hospital Regensburg, 93053 Regensburg, Germany
2 Department of Conservative Dentistry and Periodontology, University Hospital Regensburg, 93053 Regensburg, Germany
3 Department of Microbiology and Hygiene, University Hospital Regensburg, 93053 Regensburg, Germany
4 Institute for Medical Microbiology, Immunology and Hygiene University Hospital Cologne and Faculty of Medicine, University of Cologne, 50935 Cologne, Germany
* Correspondence: agnes.schroeder@ukr.de; Tel.: +49-941-944-4991
† These authors contributed equally to this work.

Supplementary Material

Uncropped Westernblots

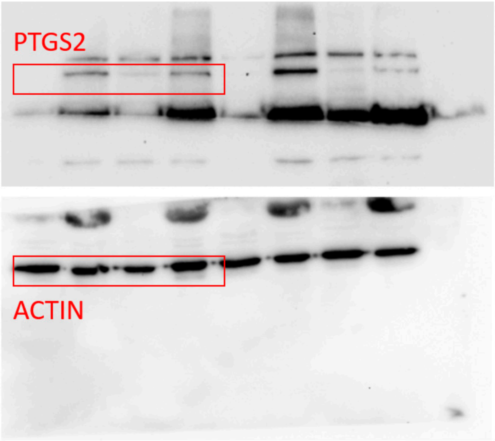

Figure S1. Uncropped Western Blot for Figure 2h.

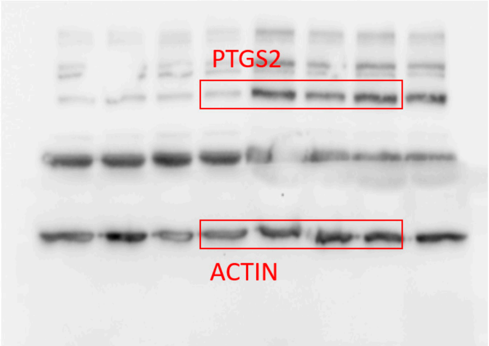

Figure S2. Uncropped Western Blot for Figure 3h.

Cell numbers and LDH release.

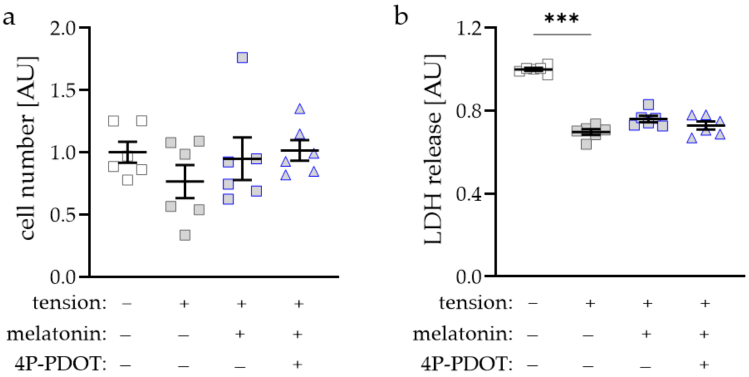

Figure S3 Impact of melatonin and tensile strain on cell number (a) and lactatdehydrogenase (LDH) release. n=6; *Statistics:* Welch-corrected ANOVA with Games-Howell multiple comparison tests; ***P < 0.001

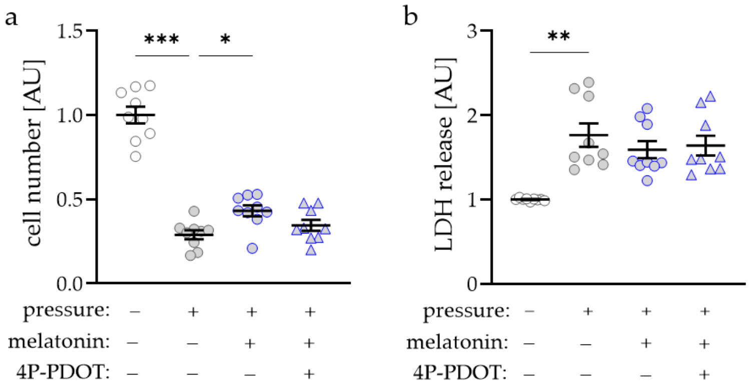

Figure S4 Impact of melatonin and compressive strain on cell number (a) and lactatdehydrogenase (LDH) release. n=9; *Statistics:* cell number: ordinary ANOVA with Holm-Sidak´s multiple comparison tests; LDH release: Welch-corrected ANOVA with Games-Howell multiple comparison tests; *P<0.05, ***P<0.001.
